# In Silico Analysis of Polycyclic Aromatic Hydrocarbon (PAH) Degrader from *Bordetella petrii* Strain P003 Isolated from Contaminated Oil of Kuwait

**DOI:** 10.3390/cimb48050527

**Published:** 2026-05-18

**Authors:** Abrar Akbar, Rita Rahmeh, Mohamed Kishk, Anisha Shajan

**Affiliations:** Kuwait Institute for Scientific Research, P.O. Box 24885, Safat 13109, Kuwait; rrahmeh@kisr.edu.kw (R.R.); mwaheed@kisr.edu.kw (M.K.); ashajan@kisr.edu.kw (A.S.)

**Keywords:** polycyclic aromatic hydrocarbons (PAHs), bioremediation, *Bordetella petrii*, in silico analysis, comparative genomics, pathway reconstruction

## Abstract

*Bordetella petrii* is an environmentally versatile Gram-negative bacterium with hydrocarbon-degrading capabilities, yet its genetic and metabolic characteristics remain poorly characterized. This study investigated the genomic features of a PAH-degrading *Bordetella petrii* strain P003 isolated from contaminated oil in Kuwait using bioinformatic approaches. The genome of *B. petrii* P003 was sequenced and analyzed for genomic islands, comparative genomics, and PAH degradation pathways. The draft genome assembly of *B. petrii* P003 was 5,011,660 bp with 49 contigs and 68.67% GC content. It contained 4687 coding sequences, 5 rRNAs, and 56 tRNAs. Prediction of genomic islands (GIs) revealed that strain P003 possessed 99 GIs, whereas the reference *B. pertii* DSM 12,804 had 58 unique GIs. Comparative genomics showed 279 locally collinear blocks with the reference strain. The P003 genome encoded multiple genes involved in PAH, naphthalene, and benzoate degradation pathways, including an 8-gene PAH operon (*pht4*, *ph2*, *pht5*, *pht3*, *pcaG*, *pcaH*, *nahAb*/*nagAb*/*ndoA*/*nbzA*). We found that *pca*G and *pca*H encode the enzymes responsible for the breakdown of PAH, protocatechuate 3,4-dioxygenase, alpha and beta subunits (EC: 1.13.11.3). The genomic analysis of *B. petrii* P003 provides insights into its PAH degradation capabilities and potential for bioremediation applications. The strain possesses an expanded repertoire of aromatic compound degradation genes compared to reference strains, suggesting enhanced metabolic versatility for degrading environmental pollutants.

## 1. Introduction

Polycyclic aromatic hydrocarbons (PAHs) are complex organic substances with interconnected rings [1]. Their primary source is the incomplete combustion or pyrolysis of organic elements, such as fossil fuels, biomass, and petroleum derivatives [2]. PAHs are ubiquitous environmental pollutants that contaminate ecosystems such as soil, water, aged oil, and air [3,4]. Owing to their long-lasting presence and harmful nature, PAHs pose considerable environmental and human health risks. A significant hurdle in the biodegradation of PAHs is the limited bioavailability of these pollutants to the degrading microorganisms [5]. Recently, there has been a growing focus on devising effective and sustainable methods for cleaning PAH-polluted sites [6]. Bioremediation, a technique that uses the metabolic abilities of microorganisms to decompose or alter organic pollutants, has emerged as a viable solution [7]. Microorganisms possess various enzymatic systems capable of converting complex PAH structures into simpler and less hazardous compounds.

*Bordetella petrii,* a Gram-negative bacterium of the genus *Bordetella*, has been identified in multiple environmental elements, such as soil, water, and animal respiratory systems [8]. This bacterium is primarily recognized for its association with respiratory illnesses in animals; however, recent research has indicated its potential role in environmental activities, such as hydrocarbon breakdown [9]. Nevertheless, the ability of *B. petrii* to break down PAHs has not been well studied, and further insights are needed concerning its genetic and metabolic strategies that may contribute to its biodegradation potential [10]. Our previous research found that strain P003 of *B. petrii* could thrive on phenanthrene and fluorene [11]. Therefore, we conducted an in silico in-depth analysis of *B. petrii* P003 in the current study. Microorganisms extracted from crude oil sludge are biodegradable, and bioremediation methods are cost-effective, eco-friendly, and adaptable [12]. Few reports exist in the literature on the degradation of compounds by the genus *Bordetella*. *Bordetella petrii* has been shown to degrade naphthalene and toluene [13]. We utilized sophisticated bioinformatics software tools to decipher its genomic characteristics through genome sequencing, comparative genomics, genomic island prediction, and pathway reconstruction models to discover the genetic and metabolic pathways related to PAH degradation in this isolate.

Understanding the genetic factors and metabolic processes involved in the breakdown of PAH by *B. petrii* serves several purposes. First, it enhances our basic understanding of microbial degradation capabilities and enriches our understanding of the variety of PAH-degrading organisms present in the environment. Second, it offers a glimpse of the genetic adaptations and evolution of PAH degradation pathways in *B. petrii*. Third, this knowledge can inform the creation of specific bioremediation plans using the inherent capabilities of *B. petrii* for the elimination and detoxification of PAHs in polluted environments. Using in silico analysis, we aimed to identify potential PAH degradation genes, anticipate their functional attributes, and outline the metabolic processes involved in PAH degradation in the *B. petrii* strain sourced from Kuwait. This research serves as a foundation for future experimental studies, setting the stage for understanding the biodegradation potential of *B. petrii* and its possible use in cleaning PAH-polluted environments. The findings emphasize the importance of computational methodologies in revealing the genetic and metabolic underpinnings of the breakdown of microbial contaminants. The results of this study are significant for the formulation of novel bioremediation tactics to mitigate crude oil toxicity.

## 2. Materials and Methods

### 2.1. Growth of Bordetella petrii Strain P003 on Hydrocarbon as the Only Source of Carbon

To assess the bacterial biodegradation capability, both phenanthrene and fluorine were used as the sole carbon source. *Bordetella petrii* strain P003 growth was monitored using Bushnell–Haas minimal agar media supplemented with 250 mg/mL phenanthrene or fluorine. Cultures were grown at 30 °C with agitation for 7 days. The growth curve pattern was determined using the viable cell count and optical density reading at OD600 (spectrophotometer). Every 24 h, samples were taken directly to measure OD600 or diluted serially and seeded to determine a viable colony-forming unit (CFU) count.

### 2.2. Genome Sequencing of the Bordetella petrii Strain P003 and Analysis of Genome Characteristics

Genomic DNA extraction and genome sequencing of *B. petrii* P003 were performed as described by Akbar et al. [11]. Assembly statistics were generated using the code from the ‘Assemblathon 2’ project [14]. An in-house R script was used to check for potential circularity in the contigs. Prokka version 1.14.53 was used to annotate the draft genomes based on functional characteristics [15].

### 2.3. Phylogenetic Tree Construction

Dendrograms were generated by neighbor-joining with the Kimura-2 parameter using 500 bootstrap replicates and MEGA XI (v12.1) software for selected strains [16].

### 2.4. Genomic Islands Prediction

In this study, genetic islands were predicted using IslandViewer v4, which includes algorithms such as Integrated, IslandPick, SIGI-HMM, IslandPath-DIMOB, and Islander, which calculate non-GI and GIs generated from related organisms [17]. Additionally, the software identifies genes associated with pathogen virulence, antimicrobial resistance, and antibiotic resistance. The provided datasets allowed us to identify how the selected genomes differed.

### 2.5. Comparative Genomics

Based on their shared and distinguishing characteristics, three previously sequenced B. petrii strains were selected for a comparative genome study (Table 1). The selected species included *B. petrii* DSM 12,804 (NC_010170), J51 (NZ_JAEP00000000.1), and J49 (NZ_JAEJ00000000.1). To achieve numerous genome alignments, we used the MAUVE (v2.4.0) plugin [18] in Geneious Prime^®^ software version 2019.2.1 [19].

### 2.6. Identification of PAH-Degrading Genes and Pathway Analysis

The PAH operon of our native strain was compared with that of the reference strain *B. petrii* DSM 12,804. The KEGG database was used to analyze the pathways involved in PAH breakdown [20].

### 2.7. Accession Number of the Genome Sequence

The annotated genome sequence of *Bordetella petrii* P003 was submitted under NZ_JAUDJE010000001 to JAUDJE0100000049 (Project ID: SAMN35827802).

## 3. Results

### 3.1. Growth of Bordetella petrii Strain P003 on Hydrocarbon as the Only Source of Carbon

**Figure 1:**
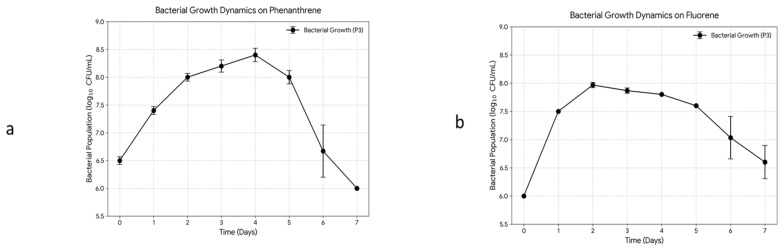
Growth of *B. petrii* strain P003 on (**a**) phenanthrene and (**b**) fluorene.

*Bordetella petrii* strain *P003* showed good polycyclic aromatic hydrocarbon degradation, especially when grown on phenanthrene and fluorene as its sole carbon and energy source (Figure 1). The growth curve demonstrated better growth on phenanthrene when compared to fluorene. Such results support the ability of this bacterium to utilize complex PAHs.

### 3.2. Genomic Features of the Bordetella petrii Strain P003

**Figure 2:**
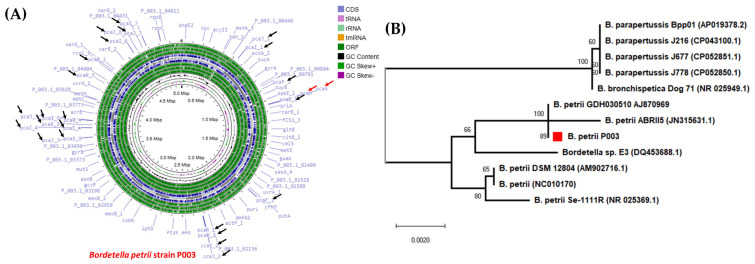
Circular genomic view of *Bordetella petrii* strain P003 representing the genes involved in PAH degradation (**A**) and the phylogenetic view of the 16S rRNA gene of strain P003 (red box) (**B**).

Through a careful genome sequencing procedure, we discerned that the genetic composition of the *B. petrii* strain P003 contained 49 unique contigs, culminating in an approximate total genome size of 5,011,660 base pairs (bp). The largest sequenced segment was 508,584 bp. The P003 genome sequence had a remarkable 68.67% GC content, suggesting potential evolutionary adaptations unique to this strain. Furthermore, the genome sequence incorporates an exhaustive set of 4687 coding sequences (CDSs), 5 ribosomal RNAs (rRNAs), and 56 transfer RNAs (tRNAs), all of which collectively govern protein synthesis and regulation within the organism. Black, green, purple, and red colors represent the GC content, GC skew +/−, and comprehensive antibiotic resistance database (CARD), respectively, in Figure 2A. The genome map of B. petrii strain P003 (Figure 2A) revealed the presence of multiple gene clusters involved in the degradation of aromatic compounds, notably the pca gene clusters (e.g., pcaA, pcaB, pcaC, pcaD, pcaG, and pcaH), which are essential components of the β-ketoadipate pathway. These genes were distributed across the genome and are indicated by directional arrows, suggesting that this strain adapted to environmental conditions involving aromatic substrates. Average nucleotide identity (ANI) analysis revealed that this strain has a notable 99.17% homology with the reference genome of Bordetella petrii GCF002261345.1, indicating a close genetic correlation and possibly equivalent functions in the two strains.

Phylogenetic analysis based on 16S rRNA sequences (Figure 2B) showed that *B. petrii* strain P003 formed a distinct subclade closely related to *B. petrii* ABRII5 and GDH030510, with a bootstrap support of 89%, suggesting close evolutionary relatedness. However, this subclade was separate from other reference *B. petrii* strains, such as DSM 12,804 and NC010170, indicating genetic divergence within the species. This finding points to possible niche-specific adaptation or horizontal gene transfer events in the strain P003. However, it is important to note that species within the *Bordetella* genus, including *B. petrii*, *B. parapertussis*, and *B. bronchiseptica*, showed significant homology, indicating their close evolutionary relationship. An extensive investigation of the P003 strain, including whole-genome analysis and phenanthrene degradation tests, provided insights into the intrinsic bacterial processes of this strain. Regarding genomic characteristics, the GC content of the P003 strain was compared with that of the reference genome *B. petrii* DSM 12,804 (Accession Number AM902716).

### 3.3. Genomic Island (GIs) Prediction in the B. petrii P003 Strain

**Figure 3:**
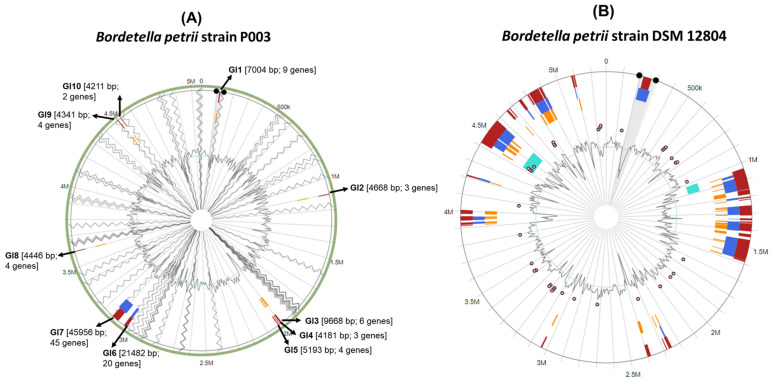
Genomic island (GI) regions in (**A**) *B. petrii* P003 and (**B**) DSM 12,804 strains predicted by integrated (red), IslandPath-DIMOB (blue), SIGI-HMM (orange), and islander (aqua).

In strain P003, ten GIs (GI1–GI10) were identified, ranging in size from 4181 to 45,956 bp, as shown in Figure 3. These islands were dispersed throughout the genome and collectively encoded various genes, many of which are associated with metabolic pathways, stress responses, and potential adaptive functions. Notably, GI7 was the largest genomic island (45,956 bp) and contained 45 genes, suggesting a significant gene acquisition event. Other prominent islands included GI6 (21,482 bp; 20 genes) and GI1 (7004 bp; 9 genes). The GC skew pattern showed characteristic deviations in these regions, supporting their foreign origin.

In contrast, *B. petrii* strain DSM 12,804 exhibited a markedly more complex pattern of genomic plasticity, with numerous diverse horizontally acquired regions scattered across the genome. These regions were visually represented as dense clusters of color-coded blocks corresponding to various functional gene categories and mobile genetic elements, including transposases and phage-related genes. The genomic content of DSM 12,804 suggests extensive horizontal gene transfer activity, potentially enhancing its environmental adaptability. The widespread distribution of these regions, particularly near the 500 kb, 1.5 Mb, and 4 Mb loci, underscores the dynamic nature of its genome compared to the relatively more streamlined acquisition observed in strain P003.

### 3.4. Identification of PAH-Degrading Genes in the Genome of B. petrii Strain P003

**Figure 4:**
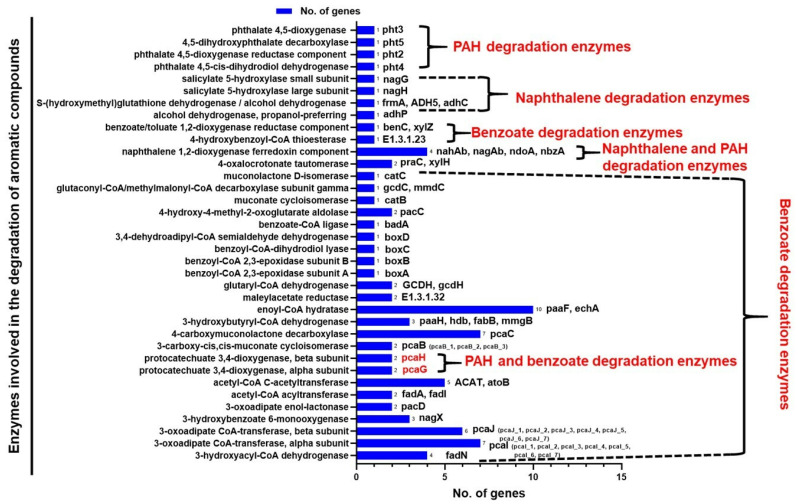
The central metacleavage pathway involved in the degradation of aromatic compounds of the *B. petrii* P003 strain.

Functional annotation and comparative analysis of genes involved in the degradation of aromatic compounds in *Bordetella petrii* strain P003 revealed the presence of a wide array of catabolic enzymes associated with the breakdown of polycyclic aromatic hydrocarbons (PAHs), naphthalene, and benzoate (Figure 4). A total of 77 genes encoding enzymes associated with aromatic compound degradation were identified. The highest gene abundance was observed for *paaF* and *echA* (10 copies), which are involved in the phenylacetyl-CoA (PAA) pathway, which plays a central role in benzoate catabolism. Several other key enzymes involved in benzoate degradation, such as *paaH*, *hdb*, *fabB*, and *mmgB* (nine genes), as well as *pcaC* (seven genes), *pcaB*, *pcaH*, *pcaA*, and *pcaG* (ranging from two to three genes each), were also notably present, indicating the metabolic versatility of the strain in utilizing aromatic substrates.

In addition to benzoate-related pathways, the genome encodes several enzymes associated with PAH and naphthalene degradation, including *pht3*–*pht5*, *nagG*, *nagH*, *frmA*, *ADH5*, *adhC*, and *adhP*. The identification of multiple gene copies, such as *pcaJ* (six copies) and *pcaI* (seven copies), further supports the potential of this organism for environmental adaptation and bioremediation through the mineralization of complex aromatic pollutants. Notably, the presence of shared enzymes such as *pcaH* and *pcaG*, which function in both PAH and benzoate degradation, highlights the metabolic overlap and integration of different aromatic degradation pathways in *B. petrii* P003. Collectively, these findings suggest that *B. petrii* P003 possesses a robust genetic repertoire for aromatic compound degradation, positioning it as a potential candidate for environmental detoxification and bioremediation strategies.

**Figure 5:**
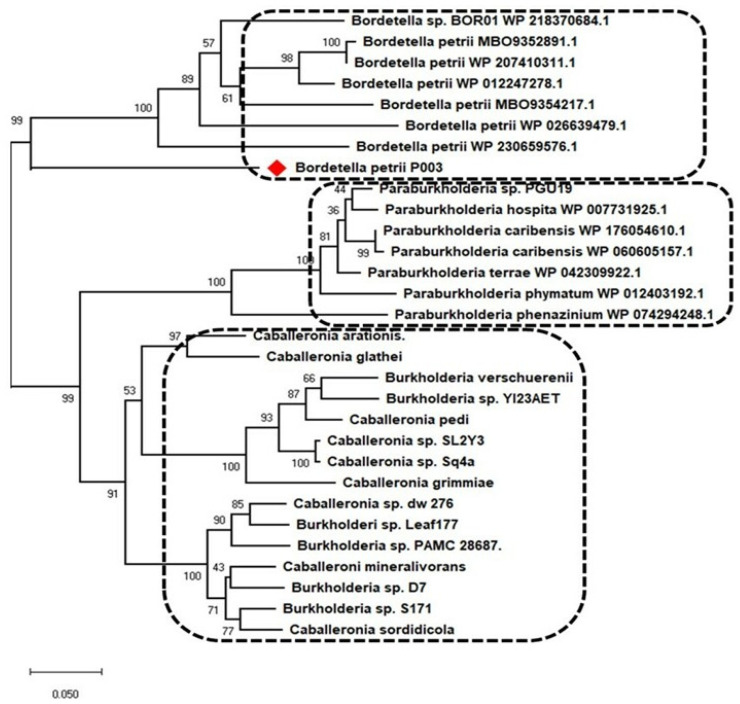
Phylogenetic tree of *B. petrii* P003 (red square represents the bacterium under study).

Phylogenetic analysis based on whole-genome sequence alignment revealed the taxonomic placement of *Bordetella petrii* strain P003 within the *Bordetella* genus (Figure 5). The maximum-likelihood tree demonstrated that strain P003 clustered tightly with other *B. petrii* reference strains, including WP_230659576.1, WP_026639479.1, WP_09354217.1, and WP_012247278.1, supported by high bootstrap values (ranging from 61 to 100), indicating a phylogenetic relatedness. The *Bordetella* clade formed a distinct and well-supported monophyletic group separate from other genera, such as *Paraburkholderia*, *Caballeronia*, and *Burkholderia*. Within this broader phylogenetic framework, *Paraburkholderia* species formed a closely related sister clade with moderate bootstrap support (44–100), followed by distinct branches comprising *Caballeronia* and *Burkholderia* taxa. The position of strain P003 (marked by a red diamond) within the *B. petrii* cluster confirmed the high degree of evolutionary conservation within the species. These findings further corroborate the genetic relatedness of strain P003 to environmental *B. petrii* isolates, supporting its placement in the *Bordetella* genus and suggesting that they have similar ecological and functional characteristics.

### 3.5. Comparison of B. petrii Genomes

**Figure 6:**
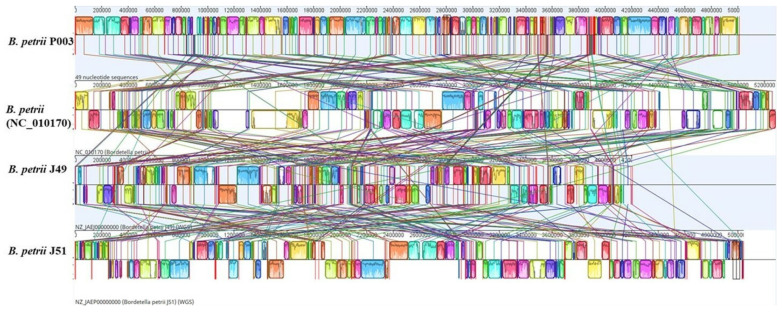
Comparative genome analysis between the genomes of *B. petrii* P003, DSM 12,804 (NC_010170), J49, and J51.

Comparative genomic synteny analysis of *Bordetella petrii* strain P003 and three reference strains (*B. petrii* NC_010170, J49, and J51) revealed a high degree of conserved gene order and structural organization, with several regions of syntenic blocks observed across the genomes (Figure 6). The isolate P003 exhibited extensive genome collinearity with *B. petrii* NC_010170, indicating close evolutionary relatedness and conservation of the gene content. However, certain genomic regions showed clear rearrangements, inversions, and insertions, particularly when compared with strains J49 and J51, as evidenced by the crossing lines and the disrupted syntenic blocks. These structural variations suggest genomic plasticity and possible strain-specific adaptation mechanisms. Additionally, the observed variability in the genome architecture among *B. petrii* strains may be attributed to the acquisition or loss of mobile genetic elements or genomic islands. The conserved core regions shared across all four strains support a common evolutionary lineage, whereas the divergent regions indicate the influence of horizontal gene transfer and selective pressure in shaping strain-specific functional capabilities.

A detailed comparative genomic analysis of the *pca* gene cluster, which is a key component of the β-ketoadipate pathway involved in the degradation of aromatic compounds, was conducted for *Bordetella petrii* strain P003 against the reference genome (NC_010170) (Table 2). In strain P003, a remarkable expansion and diversification of *pca* genes was observed. Specifically, the genome encodes at least seven distinct paralogs of *pcaI* and *pcaJ*, which encode subunits A and B of 3-oxoadipate CoA-transferase (EC 2.8.3.6, COG1788 and COG2057, respectively), critical enzymes for converting β-ketoadipate into its CoA-activated form. These gene copies are distributed across various locally collinear blocks (LCBs), suggesting multiple duplications or horizontal gene transfer events, and their consistent reverse orientation may indicate conservation in transcriptional directionality.

In addition to *pcaI* and *pcaJ*, strain P003 harbors three variants of *pcaF* (beta-ketoadipyl-CoA thiolase, EC 2.3.1.174), which is involved in the cleavage of β-ketoadipyl-CoA, a step downstream in the pathway. Furthermore, multiple copies of *pcaR* (COG1414), encoding transcriptional regulators of the *pca* regulon, were found, indicating potential regulatory complexity that might allow fine-tuning of responses to different aromatic substrates. The presence of *pcaG* and *pcaH*, which encode the alpha and beta subunits of protocatechuate 3,4-dioxygenase (EC 1.13.11.3), was conserved between P003 and the reference strain, highlighting the importance of ring-cleavage steps in protocatechuate catabolism.

Interestingly, several *pcaB* genes (encoding 3-carboxy-cis,cis-muconate cycloisomerase, EC 5.5.1.2) were also found, some of which lie outside the LCBs, potentially representing genomic islands or mobile elements. Moreover, the reference strain contained unique genes, such as *pcaQ*, another regulatory gene, and two different variants of *pcaD* (3-oxoadipate enol-lactonase, EC 3.1.1.24), which were not observed in the same configuration in strain P003. The redundancy and widespread genomic distribution of *pca* gene clusters in P003 strongly suggest a high degree of metabolic plasticity and environmental adaptability, enabling the organism to degrade a broad spectrum of aromatic compounds. This gene organization may reflect adaptation to complex ecological niches, where the availability and types of aromatic substrates fluctuate, thus providing a selective advantage for survival and ecological competitiveness.

## 4. Discussion

This study focused on the biochemical basis of polycyclic aromatic hydrocarbon (PAH) degradation in bacteria and the discovery of a novel PAH-degrading bacterium. In silico characterizations of such bacteria have been performed to deepen our understanding of PAH-degrading bacteria. *Bordetella petrii* strain P003 was isolated from crude oil in Kuwait. Based on biochemical tests and the 16S rRNA phylogenetic tree, it was classified as a member of the *Bordetella* genus. This strain can biodegrade phenanthrene (3-ring), pyrene (4-ring), and benzo[a]pyrene (5-ring) PAHs [11]. Numerous bacterial species, including *Bordetella*, *Stenotrophomonas*, *Pseudomonas*, and *Rhodococcus* spp., have been found to possess the ability to degrade PAHs. These bacteria can survive in specific ecological niches with limited nutrient availability and extreme environmental conditions, such as high acidity or alkalinity [21,22]. Intriguingly, the genome size of our local isolate was found to be smaller than that of the DSM 12,804 strain, the latter displaying a genome size of 5,287,950 bp, a GC content of 65.48%, 5105 genes, 9 rRNAs, and 51 tRNAs [9]. Our current research isolated *B. petrii* P003 from oil-contaminated soil samples, indicating its potential to produce PAH-degrading enzymes and survive in various extreme environmental conditions.

*Bordetella petrii* has several distinctive genomic islands (GIs) identified through various predictive analyses. GIs in *B. petrii* can be predicted using tools such as IslandViewer, SIGI-HMM, and IslandPath-DIMOB, which rely on features such as atypical GC content, gene presence for mobility, and codon usage bias [17,23]. The genome contains several laterally acquired genomic islands (GI1–GI7) that display characteristics typical of mobile elements, including integrase genes and direct repeats. Genomic islands (GIs) contribute significantly to the evolutionary process of bacteria by incorporating novel characteristics, such as virulence attributes and antibiotic resistance genes. Regarding *Bordetella* sp., numerous research initiatives have recognized and detailed GIs across various species within this genus [24,25]. The investigation led by Parkhill et al. [26] compared genome sequences of *Bordetella pertussis*, *Bordetella parapertussis*, and *Bordetella bronchiseptica*. This scrutiny revealed the existence of distinctive GIs attached to each pathogen. Certain GIs were common between *B. pertussis* and *B. parapertussis*, indicating the possible occurrence of HGT among these intricately connected species. Such comparative genomic studies facilitate our understanding of the evolutionary behavior patterns of different *Bordetellae* species’ GIs. A study by Gross et al. [9] observed the presence of seven GIs in the *B. petrii* strain DSMZ12804, where four GIs (namely, GI1, GI2, GI3, and GI6) were closely related to the element *clc* of *Pseudomonas knackmusii* B13, which is involved in the biodegradation of chloroaromatic compounds. Furthermore, GI4 is similar to Tn4371 (a conjugative transposon) that carries genes involved in biphenyl degradation. The presence of these GIs highlights *B. petrii*’s metabolic versatility and its capability to adapt to environmental changes, as it can utilize a range of aromatic compounds [27]. The genomic structure reflects a mosaic genome due to extensive HGT, providing insights into the evolutionary dynamics of the *Bordetella* genus. However, during our examination, considerable deviation was observed in the GIs among the various strains of *B. petrii* analyzed.

In a recent study, Harris et al. unveiled the initial genetic blueprint of *Bordetella* sp. FB-8 was obtained from a uranium mining region in Germany. This site contains a diverse range of hydrocarbons, including PAHs [28]. Interestingly, these bacteria can break down these compounds, which is promising for bioremediation procedures. Genomic analysis suggested the presence of specific genes and metabolic pathways associated with PAH decomposition in this strain. Gross et al. [9] revealed genes that contribute to PAH breakdown in *Bordetella* sp. and other environmental bacterial species. They aimed to understand the genetic underpinnings of PAH degradation while deciphering how these bacteria can exploit PAHs as a carbon source. Our current study highlights the genetic capacity of *B. pertii* P003 to metabolize PAHs, providing insights into its environmentally adaptive nature and potential bioremediation capabilities. Interestingly, Abo-State et al. [29] later discovered that certain strains within the *Bordetella* genus exhibited metabolic proficiencies, enabling them to use these environmental contaminants as energy and carbon sources owing to specific gene codes capable of degrading PAHs. This capability could offer a competitive edge in PAH-contaminated environments, promoting their persistence and proliferation in such ecosystems [22]. A more thorough understanding of the genetic basis of PAH degradation in *Bordetella* sp. could yield significant insights for bioremediation approaches, as these bacteria may be leveraged to decontaminate areas polluted with PAHs. This finding aligns with numerous studies that have identified functional genes within PAH-decomposing organisms belonging to the genera *Pseudomonas* and *Rhodococcus* [30].

A study by Parke in 1996 found evidence that structural genes *pca* in *Agrobacterium tumefaciens* are arranged in the order *pcaDCHGB*, with the *pcaIJ* gene independently regulated by α-ketoadipate [31]. Subsequent work by Parke [32] found that *pcaQ*, a regulatory gene located upstream of *pcaD*, functions in trans to activate the expression of enol-lactone hydrolase (*pcaD*). The process described here is present in *B. petrii* DSM 12804. The breakdown of PAHs, cyclic hydrocarbons, and other aromatic compounds involves a variety of enzymes, including oxidoreductases, hydroxylases, dehydrogenases, and dioxygenases [33]. The significant presence of genes responsible for the degradation of aromatic compounds in *B. petrii* P003 may be a distinguishing characteristic of this bacterium, which originates from oil-contaminated environments. It has been demonstrated that enzymes and proteins that biodegrade PAHs can be produced in larger quantities, increasing the energy gain of these bacteria and their survival rate, making them more able to adapt [34].

A study by Wang et al. [35] suggested that ring-hydroxylated dioxygenase (RHD) enzymes are involved in the degradation of benzo(a)pyrene and other polycyclic aromatic hydrocarbons. This finding is consistent with that of a previous study that found a high abundance of genes involved in the degradation of aromatic hydrocarbons, such as dioxygenases, in oil-contaminated areas [36]. The differences found in the genome of *B. petrii* P003 compared to other strains of *Bordetella* or other biodegraders might explain how this organism can efficiently degrade PAHs and other xenobiotics. Notably, the dimethylformamide (DMF) degradation pathway has been described in several *Bordetella* and *Parabordetella* species. This pathway involves three enzymatic steps: (1) hydrolysis of DMF to formate and dimethylamine, (2) oxidation of dimethylamine to formaldehyde, and (3) assimilation of formaldehyde into central metabolism [34]. Analysis revealed a system of PAH degradation with eight genes highly similar to *naBAF* genes, as well as other genes necessary for the conversion of naphthalene to salicylate. Other PAH dioxygenases have been characterized in mycobacteria, which induce the degradation of PAHs with side-chain dioxygenases that cause ring opening. Furthermore, genome analysis revealed the presence of genes involved in the transfer of intermediate metabolites from aromatic compound metabolism to central metabolism. This suggests that *B. petrii* P003 has complete pathways for the degradation and utilization of aromatic compounds. Identifying these genes in the genome provides additional evidence for the genetic basis of *B. petrii* P003’s ability to utilize carbon sources derived from aromatic oils. This work represents genomic predictions by growth phenotypes only, with no direct measurements of degradation efficiency.

## 5. Conclusions

The genomic analysis of *Bordetella petrii* strain P003 reveals a genome size of approximately 5,011,660 base pairs with a high GC content of 68.67%, indicating potential evolutionary adaptations. The genome contains multiple gene clusters involved in aromatic compound degradation, particularly the *pca* gene clusters, suggesting adaptation to environments with aromatic substrates. Phylogenetic analysis places *B. petrii* strain P003 in a distinct subclade closely related to other *B. petrii* strains, but with some genetic divergence. Ten genomic islands were identified, encoding various genes associated with metabolic pathways, stress responses, and potential adaptive functions. The strain possesses 77 genes encoding enzymes for aromatic compound degradation, including PAH, naphthalene, and benzoate degradation pathways, positioning it as a potential candidate for bioremediation strategies. Comparative genomic analysis revealed high synteny with other *B. petrii* strains but also showed evidence of genomic plasticity. The *pca* gene cluster in strain P003 showed remarkable expansion and diversification compared to the reference genome, suggesting enhanced metabolic plasticity and environmental adaptability. The redundancy and widespread genomic distribution of *pca* gene clusters indicate adaptation to complex ecological niches with varying availability and types of aromatic substrates. These genomic features suggest that *B. petrii* strain P003 has evolved significant capabilities for environmental adaptation and aromatic compound degradation.

## Figures and Tables

**Table 1:** Genome features of different *Bordetella petrii*.

*B. petrii* Strains	Genome Size	Accession No.	Genes	CDSs	rRNA	tRNA	GC %
P003	5,011,660	NZ_JAUDJE010000000	4793	4737	5	47	68.5%
DSM 12804	5,287,950	NC_010170	5111	5047	9	51	65.5%
J51 or ASM51896v1	5,043,350	NZ_JAEP00000000.1	4877	4788	8	47	68.5%
J49	4,204,974	NZ_JAEJ00000000.1	3990	3934	5	47	65.5%

**Table 2:** Comparison of *pca* genes among the selected genomes.

Gene ID	Length_bp	EC_Number	COG	Product	LCB No.	Strain (Locations Co-Ordinates)	Orientation
pcaI_1	693	2.8.3.6	COG1788	3-oxoadipate CoA-transferase subunit A	LCB 85	P003 (bases 2,028,181 to 1,919,384)	reverse
pcaJ_1	678	2.8.3.6	COG2057	3-oxoadipate CoA-transferase subunit B	LCB 85	P003 (bases 2,028,181 to 1,919,384)	reverse
pcaF_1	1290	2.3.1.174	-	Beta-ketoadipyl-CoA thiolase	LCB 75	P003 (bases 2,320,129 to 2,285,642)	forward
pcaG	591	1.13.11.3	COG3485	Protocatechuate 3,4-dioxygenase alpha chain	LCB 126	P003 (bases 3,129,161 to 3,121,119)	forward
pcaH	717	1.13.11.3	COG3485	Protocatechuate 3,4-dioxygenase beta chain	LCB 126	P003 (bases 3,129,161 to 3,121,119)	forward
pcaB_1	1440	5.5.1.2	COG0015	3-carboxy-cis,cis-muconate cycloisomerase	No	P003 (bases 3,129,162 to 3,133,835)	forward
pcaF_2	1206	2.3.1.174	COG0183	Beta-ketoadipyl-CoA thiolase	LCB 94	P003 (bases 4,349,587 to 4,169,679)	forward
pcaR_1	888	-	COG1414	Pca regulon regulatory protein	LCB 180	P003 (bases 4,729,235 to 4,653,448)	forward
pcaI_2	681	2.8.3.6	COG1788	3-oxoadipate CoA-transferase subunit A	LCB 180	P003 (bases 4,729,235 to 4,653,448)	reverse
pcaJ_2	657	2.8.3.6	COG2057	3-oxoadipate CoA-transferase subunit B	LCB 180	P003 (bases 4,729,235 to 4,653,448)	reverse
pcaR_2	828	-	COG1414	Pca regulon regulatory protein	LCB 180	P003 (bases 4,729,235 to 4,653,448)	reverse
pcaI_3	687	2.8.3.6	COG1788	3-oxoadipate CoA-transferase subunit A	LCB 259	P003 (bases 1,555,641 to 1,429,089)	reverse
pcaJ_3	663	2.8.3.6	COG2057	3-oxoadipate CoA-transferase subunit B	LCB 259	P003 (bases 1,555,641 to 1,429,089)	reverse
pcaI_4	669	2.8.3.6	COG1788	3-oxoadipate CoA-transferase subunit A	LCB 235	P003 (bases 1,627,715 to 1,611,328)	reverse
pcaJ_4	642	2.8.3.6	COG2057	3-oxoadipate CoA-transferase subunit B	LCB 235	P003 (bases 1,627,715 to 1,611,328)	reverse
pcaR_3	762	-	COG1414	Pca regulon regulatory protein	LCB 236	P003 (bases 1,627,766 to 1,651,262)	reverse
pcaJ_5	645	2.8.3.6	COG2057	3-oxoadipate CoA-transferase subunit B	LCB 112	P003 (bases 1,681,324 to 1,688,444)	reverse
pcaI_5	690	2.8.3.6	COG1788	3-oxoadipate CoA-transferase subunit A	LCB 112	P003 (bases 1,681,324 to 1,688,444)	reverse
pcaR_4	831	-	COG1414	Pca regulon regulatory protein	LCB 112	P003 (bases 1,681,324 to 1,688,444)	forward
pcaR_5	771	-	COG1414	Pca regulon regulatory protein	LCB 261	P003 (bases 2,547,998 to 2,449,394)	reverse
pcaB_2	1341	5.5.1.2	COG0015	3-carboxy-cis,cis-muconate cycloisomerase	LCB 271	P003 (bases 2,548,003 to 2,644,222)	reverse
pcaJ_6	681	2.8.3.6	COG2057	3-oxoadipate CoA-transferase subunit B	LCB 226	P003 (bases 2,828,280 to 2,843,757)	reverse
pcaI_6	681	2.8.3.6	COG1788	3-oxoadipate CoA-transferase subunit A	LCB 226	P003 (bases 2,828,280 to 2,843,757)	reverse
pcaJ_7	741	2.8.3.6	COG2057	3-oxoadipate CoA-transferase subunit B	LCB 260	P003 (bases 2,991,416 to 2,951,991)	forward
pcaI_7	666	2.8.3.6	COG1788	3-oxoadipate CoA-transferase subunit A	LCB 260	P003 (bases 2,991,416 to 2,951,991)	forward
pcaB_3	1371	5.5.1.2	COG0015	3-carboxy-cis,cis-muconate cycloisomerase	No	P003 (bases 3,567,495 to 3,584,759)	reverse
pcaF	1206	2.3.1.174	COG0183	Beta-ketoadipyl-CoA thiolase	LCB 94	NC_010170 (bases 2,939,721 to 2,768,258)	forward
pcaD	780	3.1.1.24	-	3-oxoadipate enol-lactonase	LCB 112	NC_010170 (bases 4,795,437 to 4,788,460)	reverse
pcaG	591	1.13.11.3	COG3485	Protocatechuate 3,4-dioxygenase alpha chain	LCB 126	NC_010170 (bases 305,513 to 275,034)	forward
pcaH	717	1.13.11.3	COG3485	Protocatechuate 3,4-dioxygenase beta chain	LCB 126	NC_010170 (bases 305,513 to 275,034)	forward
pcaQ	915	-		Pca regulon regulatory protein	LCB 126	NC_010170 (bases 305,513 to 275,034)	reverse
pcaD	813	3.1.1.24	-	3-oxoadipate enol-lactonase	LCB 229	NC_010170 (bases 1,696,170 to 1,331,650)	forward

## Data Availability

The genome described in this study is submitted to the National Bank of Genome under accession number NZ_JAUDJE010000000.

## Abbreviations

PAHs	Polycyclic aromatic hydrocarbons
GIs	Genomic Islands
LCBs	Local collinear blocks
KEGG	Kyoto Encyclopedia of Genes and Genomes
bp	base pairs
ANI	Average nucleotide identity
CDS	Coding sequence
HGT	Horizontal gene transfer
ORFs	Open reading frame
